# Distinct Spatiotemporal Activation Patterns of the Perirhinal-Entorhinal Network in Response to Cortical and Amygdala Input

**DOI:** 10.3389/fncir.2016.00044

**Published:** 2016-06-14

**Authors:** Janske G. P. Willems, Wytse J. Wadman, Natalie L. M. Cappaert

**Affiliations:** Center for NeuroScience, Swammerdam Institute for Life Sciences, University of AmsterdamAmsterdam, Netherlands

**Keywords:** voltage-sensitive dye imaging, inhibitory control, acute slices, mouse, network recruitment, parahippocampal region

## Abstract

The perirhinal (PER) and entorhinal cortex (EC) receive input from the agranular insular cortex (AiP) and the subcortical lateral amygdala (LA) and the main output area is the hippocampus. Information transfer through the PER/EC network however, is not always guaranteed. It is hypothesized that this network actively regulates the (sub)cortical activity transfer to the hippocampal network and that the inhibitory system is involved in this function. This study determined the recruitment by the AiP and LA afferents in PER/EC network with the use of voltage sensitive dye (VSD) imaging in horizontal mouse brain slices. Electrical stimulation (500 μA) of the AiP induced activity that gradually propagated predominantly in the rostro-caudal direction: from the PER to the lateral EC (LEC). In the presence of 1 μM of the competitive γ-aminobutyric acid (GABA_A_) receptor antagonist bicuculline, AiP stimulation recruited the medial EC (MEC) as well. In contrast, LA stimulation (500 μA) only induced activity in the deep layers of the PER. In the presence of bicuculline, the initial population activity in the PER propagated further towards the superficial layers and the EC after a delay. The latency of evoked responses decreased with increasing stimulus intensities (50–500 μA) for both the AiP and LA stimuli. The stimulation threshold for evoking responses in the PER/EC network was higher for the LA than for the AiP. This study showed that the extent of the PER/EC network activation depends on release of inhibition. When GABA_A_ dependent inhibition is reduced, both the AiP and the LA activate spatially overlapping regions, although in a distinct spatiotemporal fashion. It is therefore hypothesized that the inhibitory network regulates excitatory activity from both cortical and subcortical areas that has to be transmitted through the PER/EC network.

## Introduction

The parahippocampal region is a cortical brain area that is involved in cognitive functions like learning and memory, object recognition, sensory representation and spatial orientation (de Curtis and Paré, 2004; de Villers-Sidani et al., 2004; Uva and de Curtis, 2005; Kealy and Commins, 2011). The parahippocampal pathway, from the perirhinal cortex (PER) to the entorhinal cortex (EC), is considered an important gateway for the cortical and subcortical information flow into the hippocampus (Burwell and Amaral, 1998; Burwell, 2000; Burwell and Witter, 2002). Although anatomical connections exist within the PER/EC network (Cappaert et al., 2014), the information transfer through this network occurs with a low probability (e.g., Biella et al., 2002; Pelletier et al., 2005; Koganezawa et al., 2008).

The PER receives input from neocortical areas such as the sensory, temporal and insular cortical areas and subcortical areas like the amygdala, basal ganglia, (hypo)thalamus, raphe nucleus and olfactory bulb (Kealy and Commins, 2011). In this study, we will compare the spatiotemporal aspect of the information propagation through the PER/EC network in response to cortical and subcortical stimulation. As representative (sub)cortical areas we will focus on the input originating in the agranular insular cortex (AiP) and the lateral amygdala (LA) as these areas are collocated with the PER/EC. The AiP integrates exteroceptive and interoceptive information (Nieuwenhuys, 2012) and the LA is crucially involved in emotion processing (Paz et al., 2006; McDonald and Mott, 2016). Both brain areas are known to project to the PER/EC network (Krettek and Price, 1977; Burwell and Amaral, 1998; Pitkänen et al., 2000; Canto et al., 2008; Kealy and Commins, 2011; Mathiasen et al., 2015). Afferents from the AiP to the PER/EC network originate in all cortical layers and consist of both glutamatergic and γ-aminobutyric acid (GABA)ergic projections, which most densely target the superficial layers of the PER and deep layers of the lateral EC (LEC) in rodents (Pinto et al., 2006; Unal et al., 2013; Mathiasen et al., 2015). All PER/EC layers receive LA afferents: PER afferents are mainly glutamatergic while projections to the EC are both GABA—and glutamatergic (Smith and Paré, 1994; Pitkänen et al., 2000; McDonald and Zaric, 2015; McDonald and Mott, 2016). These GABAergic projections together with functional evidence (Martina et al., 2001a; Biella et al., 2002; Pelletier et al., 2004; Apergis-Schoute et al., 2007) suggest that the inhibitory system plays a crucial role in the control of the information flow through the PER/EC network. Studies showed that activity evoked by PER and LA stimulation can coincide in the EC (Kajiwara et al., 2003; Koganezawa et al., 2008). However, the spatiotemporal dynamics of PER/EC network recruitment and the modulation by GABAergic the inhibition in response to AiP or LA stimulation needs to be revealed.

The current study determined the functional organization of the AiP or LA projections to the PER/EC network in horizontal mouse brain slices, at a high spatio-temporal resolution with voltage sensitive dye (VSD) imaging. VSD imaging reveals the population changes in membrane potential in these horizontal slices, which allowed a detailed analysis of the spatial and temporal pattern of network recruitment. We addressed the dynamics of the mouse PER/EC network activity in response to AiP or LA stimulation by comparing the recruitment sequence of the AiP or LA evoked activity in the network in control condition and after GABA_A_ dependent inhibition was reduced.

## Materials and Methods

### Slice Preparation, Solutions and VSD Staining

**Figure 1:**
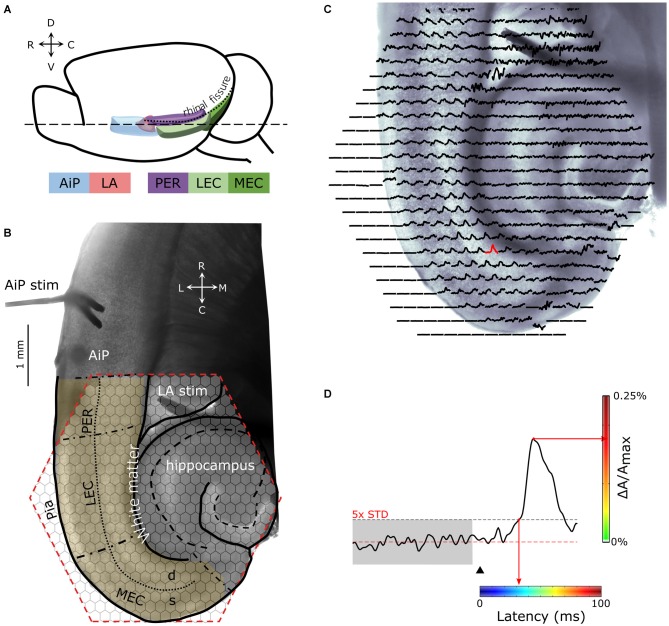
**(A)** Schematic drawing of the lateral view of the mouse brain showing the structures of interest. **(B)** Example of a 400 μm thick horizontal brain slice obtained at the level dashed line (- - -) indicated in **(A)**. Stimulation electrodes were placed in the agranular insular cortex (AiP; neocortical area) and the lateral amygdala (LA), indicated by “AiP stim” and “LA stim”, respectively. The anatomical borders of the perirhinal (PER), lateral EC (LEC), and medial EC (MEC) are indicated with black, dash-dotted lines. The border between the deep and superficial layers in the PER, LEC and MEA is indicated with a dotted line. The cell layers of the hippocampus are indicated with a dashed line. The hexagonal shaped area of the 464-channel photodiode array overlying the PER, LEC and MEC is indicated in red. Neuronal activity was recorded in the region of interest (yellow shaded region). **(C)** Spatial overview of optical recording with 464-channels of a parahippocampal—hippocampal slice preparation stained with the voltage-sensitive dye NK3630. Each trace represents membrane potential changes of a population of neurons recorded by the channel in response to stimulation in the AiP. **(D)** Example trace of the evoked response recorded in a single channel in the MEC (the red response in **C**). The arrowhead indicates the time of stimulation. The red line marks the baseline signal, the gray shaded and the black dotted line area shows the 5× SD over 100 ms of baseline, which is used as the threshold for response latency determination. For spatial representation of the response latency in every channel, the latency was converted to a color-coded scale, in which blue codes the smallest and red the largest latency. The amplitude of the signal (ΔA/A_max_) in percentage is color coded from green (0%) to red (0.25%) as indicated with the vertical colorbar to visualize the magnitude of the response at a single time point. Abbreviations: AiP, Agranular insular cortex; LA, Lateral amygdala; PER, Perirhinal cortex; LEC and MEC, Lateral and medial entorhinal cortex; d, Deep layers; s, Superficial layers; C, caudal; D, dorsal; L, lateral; M, medial; R, rostral; V, ventral.

The experiments and animal care were approved by the Animal Care and Use committee of the University of Amsterdam and were in accordance with European guidelines. Brain slices were obtained from male and female C57BL/6 mice (Harlan Netherlands BV, Horst) at postnatal day 28–42. At this age, the inhibitory system is considered as matured (Del Rio et al., 1992). Experiments were performed on 11 horizontal brain slices (Figures 1A,B) from nine unique mice, containing the AiP, LA, PER, LEC, and medial EC (MEC). The mice were decapitated, the brain was rapidly removed from the skull and stored in ice-cold artificial cerebrospinal fluid (ACSF) containing (in mM): 120 NaCl, 3.5 KCl, 5 MgSO_4_, 1.25 NaH_2_PO_4_, 2.5 CaCl_2_, 25 NaHCO_3_, and 10 glucose oxygenated with 95% O_2_/5% CO_2_ (pH 7.4). Four-hundred micrometers (400 μm) thick horizontal slices were cut (Figures 1A,B) using a VT1200S vibratome (Leica Biosystems, Nussloch, Germany). Projections from the AiP and LA towards the PER/EC network are found to be present in horizontal slices of the rodent brain (von Bohlen und Halbach and Albrecht, 2002; Kajiwara et al., 2003; Koganezawa et al., 2008; Mathiasen et al., 2015).

After acclimatization for 30 min, slices were incubated for 1 h with 0.007 mg/ml of the oxonol VSD, NK3630 (Hayashibara Biochemical Laboratories Inc., Kankoh-Shikiso, Okayama, Japan). After staining, the slices were kept at room temperature in a holding chamber on a membrane (MilliPore LCR membrane filter, FHLC02500, Polytetrafluoroethylene hydrophilic membrane with 0.45 μm pore size, Millipore, Billerica, NA, USA), placed on a well filled with ACSF in a moistened 95% O_2_/5% CO_2_ atmosphere.

### Electrical Stimulation

Electrical stimuli were applied through a bipolar stimulation electrode (100 μm diameter isolated stainless steel wire) with a tip separation of 150–200 μm. Stimulation electrodes were placed under visual guidance in the superficial layers of the AiP and in the LA (Figure 1B). Biphasic square pulses of 0.3 ms were applied through a custom-made current source with amplitudes of 50, 100, 300 and 500 μA.

### Drugs

The GABA_A_ antagonist bicuculline was bath applied at 1 μM (Tocris Bioscience, Bristol, UK). This concentration never evoked aberrant spontaneous activity in slices (Menendez de la Prida and Pozo, 2002; Kajiwara et al., 2003; Koganezawa et al., 2008).

### VSD Imaging

The electrically-evoked responses were recorded with VSD imaging. The stained slices were placed in the recording chamber mounted on a microscope (Axioskop 2 FS, Zeiss, Germany) and perfused with oxygenated ACSF of 30°C at a rate of 2 ml/min. The microscope was mounted on an isolation stage (Minus K Technology, Inglewood, CA, USA) on top of a stable marble table.

Slices were illuminated with a 100 W halogen-tungsten filament bulb, powered by a DC voltage source. The excitation light was filtered with a 705 ± 60 nm interference filter. Optical responses were recorded using a 464-channel photodiode array (H-469II Photodiode Array, WuTech Instruments, Gaithersburg, MD, USA). A 5× objective (0.25 NA Fluar, Zeiss, Wetzlar Germany) was used to project the slice onto the diode array (Figures 1B,C). The data acquisition was controlled by a custom-made program (for details, see Wu et al., 1999). The signal from each diode was digitized at 1 kHz with a 12-bit data acquisition board (DAP 3200a/415 Microstar Laboratories, Bellevue, WA, USA). A digital image of the slice was acquired (SPOT, Imaging diagnostics, Sterling Heights, MI, USA) for offline superposition of the slice morphology over the diode recording sites (Figure 1B).

Membrane depolarization is reflected by NK3630 as a decrease in light absorption (Jin et al., 2002), which is represented in our measurements as a positive signal. The changes in light absorption [ΔA(t)] are proportional to the absolute light level A. To get a relevant signal with sufficient dynamic range we recorded ΔA(t) after high-pass filtering (>0.2 Hz) with a high-gain setting (500×) and then divided this ΔA(t) recorded at each diode to its absolute light level (A_max_) that was recorded in a low gain setting after the transition from light-off to light-on. We assume that ΔA(t)/A is well approximated by ΔA(t)/A_max_. A_max_ was repeatedly determined to check and correct for possible signal degradation over the time period of the recording.

### VSD Data Analysis

Analysis of the data was performed using custom-made software in MatLab (MathWorks, Natick, MA, USA). Diode channels recording the PER and EC (Figure 1B) were selected for further analysis. Recordings of the evoked responses at each stimulus intensity, were averaged over at least three artifact-free, consecutively acquired realizations. Instrumentation offset, determined by the mean ΔA/A_max_ over a 100 ms time window before the stimulus, was subtracted from each recording. Furthermore, the recordings were filtered in space with a 2D Gaussian filter with a kernel width of one inter-diode distance (approximately 150 μm) and filtered in time using a running average filter over a 5 ms window.

Positive VSD signals mainly reflect the dendritic depolarization of neurons (Chemla and Chavane, 2010). We restricted the analysis in this study to the first positive reflection that was present after stimulation in all of our experiments. This positive reflection is hereafter referred to as “the response” (Figure 1D). The undershoot following this initial response, probably as a result of a change in intrinsic properties of the slice after activity, was not further analyzed (Shoham et al., 1999).

### Histology and DiI Tracing

After VSD imaging, slices were fixed with 4% paraformaldehyde (PFA) overnight at 4°C. Slices were stained free floating with cresyl-violet, dehydrated with alcohol, cleared in xylene, mounted and coverslipped. Borders between the PER, LEC and MEC were identified based on the anatomical and cytoarchitectonic characteristics of the different areas (Insausti et al., 1997; Burwell, 2001; Paxinos and Franklin, 2001; van Groen, 2001) and the placement of stimulation electrodes in the AiP and LA was verified.

A DiI neuronal tracing experiment was performed to examine the axonal projections originating in the AiP and LA onto the PER/EC network in slices. Sonified DiI crystals (ThermoFisher Scientific, Waltham, ME, USA) were injected into the AiP and LA of 400 μm horizontal slices containing the AiP, LA and PER/EC network. The slices were incubated in oxygenated (95%O_2_/5%CO_2_) ACSF at room temperature for 24 h. Slices were fixed in 4% PFA (30 min at RT) and stored in 30% sucrose in phosphate buffered saline for cryoprotection. For imaging, slices were snap frozen and re-sectioned into 16 μm sections. Sections were embedded in VectaShield (Vector Laboratories, Burlingame, CA, USA) containing DAPI for nuclear counterstaining.

### Statistics

All values are reported as mean and standard error of the mean (SEM). Unless otherwise mentioned, Students *t*-test or linear regression were used to perform statistical analysis in MatLab (Mathworks, Natick, MA, USA). *p* < 0.05 was considered to indicate significance of the results.

## Results

### AiP but not LA Stimulation Evokes Responses in the PER/EC Network

#### AiP Stimulation

**Figure 2:**
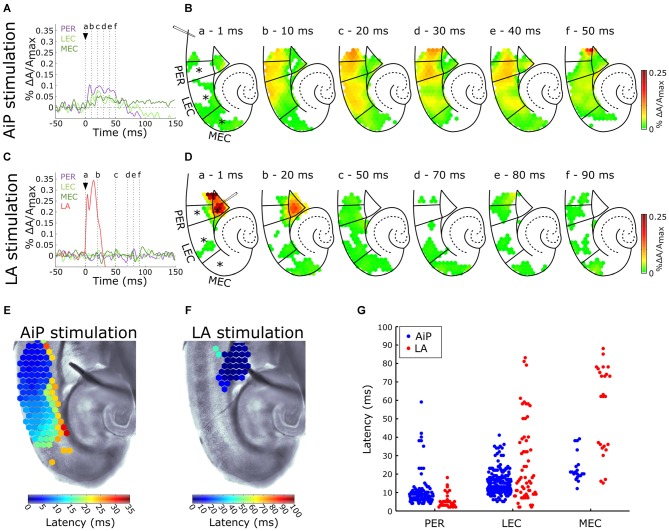
**Typical example of the temporal (A,C) and spatial (B,D) pattern of evoked AiP and LA responses at 500 μA in the subregions PER, LEC and MEC when slices were perfused with normal artificial cerebrospinal fluid (ACSF; control condition). (A,C)** Evoked response recorded at selected channels (indicated with an asterisk (*) in **B**) recorded in the PER (purple), LEC (light green), MEC (dark green) and LA (red) after AiP **(A)** or LA **(C)** stimulation. The stimulus was applied at *t* = 0 ms (black arrow head). **(B,D)** VSD signal at every channel is plotted with a color-coded scale (0–0.25% ΔA/A_max_) at specific time points **(a–f)** to visualize the spatiotemporal distribution of the evoked activity. These spatial maps show evoked response amplitude after stimulation of the AiP (**B**; at time points **(a–f)** indicated with dotted lines in **A**) and LA (**D**; at six time points **(a–f)** indicated with dotted lines in **C**). Borders of the subregions are separated by solid lines. **(E,F)** Spatial distribution of response latencies at every channel after AiP **(E)** and LA **(F)** stimulation, applied at *t* = 0, visualized using a color-coded scale (bins of 1 ms). Note that the time window of the color scale bar for AiP stimulation **(E)** is shorter than for LA stimulation **(F)**. **(G)** The latency of the responses in the PER, LEC and MEC evoked by AiP (blue dots) and LA stimulation (red dots) of obtained from all tested slices (*n* = 11). For abbreviations see Figure 1.

Control experiments were recorded in 9 out of 11 slices in standard ACSF. Figure 2A shows a typical example of the time course of the signals recorded from channels in the PER, LEC, and MEC in response to electrical stimulation (500 μA) in the superficial layers of the AiP. To visualize the spatiotemporal distribution of the evoked response, the spatial pattern of the activity in the PER/EC network at specific time points is shown in Figure 2B. The AiP-evoked response first appeared in the PER (Figures 2A,Bb), followed by a response in the LEC (Figures 2A,Bb).

To evaluate the recruitment sequence of the PER/EC network, the response latencies, defined as the time difference between the moment of stimulation and the onset of the evoked response, were determined for the responses obtained from PER, LEC and MEC. The onset of the evoked response for each diode channel was defined as the point in time where the signal amplitude exceeded 5× the standard deviation of the baseline signal determined from a 100 ms time window before the stimulation (Figure 1D; Hama et al., 2015). The typical latency pattern in response to AiP stimulation showed a gradual increase in latency from the PER towards the LEC (Figure 2E), in which PER activity preceded LEC activity (Figures 2E,G). AiP evoked activity was not conveyed to the MEC in 8/9 slices the control condition.

#### LA Stimulation

The LA was stimulated at 500 μA in the same slices to examine LA evoked neuronal activity in the PER/EC network in the control condition. The typical time course (Figure 2C) and a typical example of the spatiotemporal distribution (Figure 2D) of the LA-evoked responses in the PER/EC network and in the LA is shown. The latter showed an evoked response immediately after stimulation (Figures 2C,D) with a short latency (<10 ms; Figure 2F). The PER/EC network displayed small, short latency responses in the deep layers of PER (Figures 2C,D,F) in a subset of slices (6/9 slices). LA stimulation evoked responses in the LEC in two out of nine slices (Figure 2G).

### The AiP and LA Recruit the PER/EC Network with a Different Latency when Inhibition is Reduced

The previous results showed that afferent stimulation recruits a relatively small part of the PER/EC network in the control situation, especially in response to LA stimulation. We hypothesize that a possible explanation resides in the activation of the GABAergic inhibitory network. The PER/EC network consists of both short and long range inhibitory connections (Pinto et al., 2006; Apergis-Schoute et al., 2007; Barinka et al., 2012; Unal et al., 2013), which likely regulate activity transfer through the PER/EC. A competitive GABA_A_ antagonist was bath applied to further address role of GABAergic inhibition in the propagation of stimulus evoked activity through the PER/EC network.

#### AiP Stimulation

**Figure 3:**
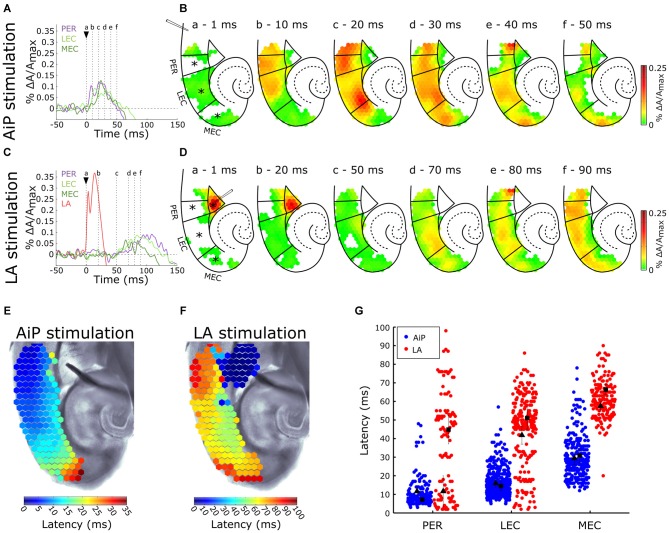
**Typical example of the temporal (A,C) and spatial (B,D) pattern of AiP and LA stimulation induced responses at 500 μA in the subregions PER, LEC and MEC in the presence of 1 μM *γ*-aminobutyric acid (GABA_A_) antagonist bicuculline. (A,C)** Evoked response recorded at selected channels (indicated with an asterisk (*) in **B**) recorded in the PER (purple), LEC (light green), MEC (dark green) and LA (red) after AiP **(A)** or LA **(C)** stimulation. The stimulus was applied at *t* = 0 ms (black arrow head). **(B,D)** VSD signal at every channel is plotted with a color-coded scale (0–0.25% ΔA/A_max_) at specific time points **(a–f)** to visualize the spatiotemporal distribution of the evoked activity. These spatial maps show the evoked response amplitude after stimulation of the AiP (**B**; at points **(a–f)** in time indicated with dotted lines in **A**) and LA (**D**; at points **(a–f)** in time indicated with dotted lines in **C**). Borders of the subregions are indicated by solid lines. **(E,F)** Spatial distribution of response latencies at every channel after AiP **(E)** and LA **(F)** stimulation, applied at *t* = 0, visualized using a color-coded scale (bins of 1 ms). Note that the time window of the color scale bar for AiP stimulation **(E)** is shorter than for LA stimulation **(F)**. **(G)** The latency of the responses in all channels in the PER, LEC and MEC evoked by AiP (blue dots) and LA stimulation (red dots) of obtained from all tested slices (*n* = 11). Every dot represents the latency on a single channel. The average latency in the superficial (squares) and the deep layers (triangles) are plotted in black. For abbreviations see Figure 1.

The AiP was electrically stimulated at 500 μA to investigate the responses in the PER, LEC, and MEC in the presence of 1 μM bicuculline in the bath. Figure 3A shows the time course of the signals from the PER, LEC, MEC and a typical example of the spatial pattern of the activity in the PER/EC network at specific time points is shown in Figure 3B. The AiP-evoked response first appeared in the PER (Figures 3A,Bb), followed by a response in the LEC (Figures 3A,Bc) and the MEC (Figures 3A,Bd–f) in all recorded slices (*n* = 11).

The latency pattern in response to AiP stimulation showed typically a gradual increase in latency from the PER towards the MEC (Figure 3E). The mean response latency of the PER/EC deep and superficial layers was determined by averaging the latencies of all channels within these three subregions (Figure 3G). The latency of the response increased from rostral to caudal in the PER/EC (*F*_(2,29)_ = 25.7, *p* < 0.001). The PER superficial and deep layers first showed activity. The LEC was activated second, first the deep layers and subsequently the superficial layers and finally the response was detected in the MEC (Figures 3A,B,G).

#### LA Stimulation

The LA was stimulated at 500 μA to examine the effect of reduced GABA_A_ mediated inhibition on evoked neuronal activity in the PER/EC network. The typical time course of evoked responses in the LA, PER, LEC, and MEC is shown in Figure 3C and a typical example of the spatiotemporal distribution of the LA stimulation evoked activity is shown in Figure 3D. The LA itself showed an evoked response directly after stimulation (Figures 3C,D). Approximately 50 ms later, the response appeared in the deep layers of the PER and EC (Figures 3C,D) and subsequently the activity was observed the superficial layers of the PER and EC.

The latency profile of the evoked responses by LA stimulation was determined to examine the pattern of PER/EC network recruitment (Figure 3F). Typically, the PER deep layers were the first to show responses, followed by the LEC and deep layers of the MEC (Figures 3F,G). The superficial layers of the PER/EC network responded later than the deep layers (Figure 3G, PER: *t*_(10)_ = 2.23, *p* = 0.05; LEC: *t*_(12)_ = 2.21, *p* = 0.047; MEC: *t*_(10)_ = 2.91, *p* = 0.02). A striking detail is that the responses in the superficial PER and EC appeared after a delay.

The latencies of AiP—and LA evoked responses were pairwise compared to address differences between the spatiotemporal pattern of the AiP and the LA evoked activity in the PER/EC network. The LA evoked response latency was longer than the AiP evoked response latency in the complete PER/EC network (Figure 3G, *t*_(8)_ = −2.58, *p* = 0.03). Thus, AiP stimulation as well as LA stimulation activated the PER/EC network if inhibition is reduced, however the onset of the recruitment of the PER/EC network is delayed for LA stimulation compared to AiP stimulation.

### Propagation of Activity Induced by AiP and LA Stimulation in the PER/EC Network

The sequence of response appearance in the PER/EC network in the presence of 1 μM bicuculline was further addressed to evaluate the propagation of activity. The position-dependent differences in latency were interpreted as an indication of propagation of activity through the PER/EC network. From the local latency values we calculated the propagation velocity of response initiation: a velocity vector that is displayed for each channel.

**Figure 4:**
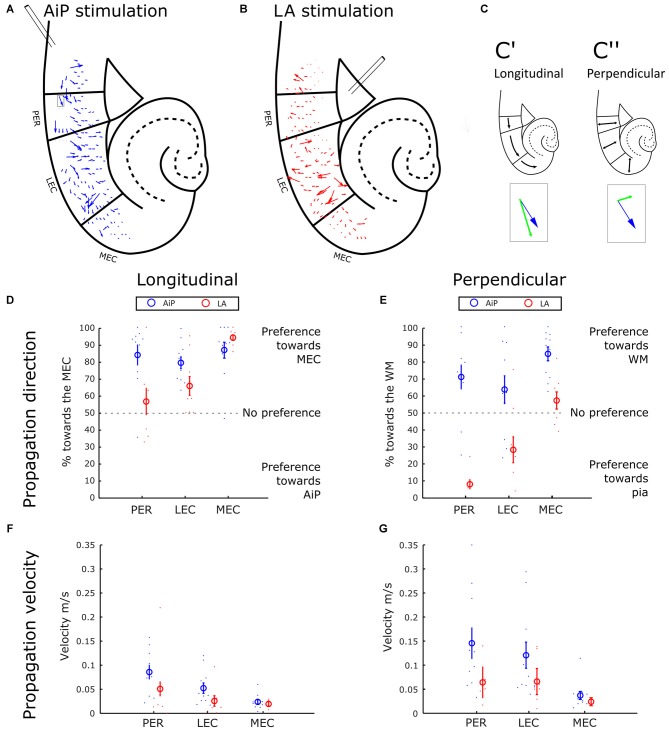
**Propagation of stimulus evoked responses. (A,B)** Typical example of the propagation velocity field in response to AiP **(A)** and LA **(B)** stimulation (500 μA) in the presence of 1 μM bicuculline. The velocity vector indicates the propagation direction and amplitude. **(C)** Example of the decomposition of a velocity vector (blue arrow indicated with the gray rectangular in A) into a longitudinal component (**C’**, green arrow in the inset) and a perpendicular component (**C”**, green arrow in the inset). **(D)** Preference of the propagation in the longitudinal direction for AiP (blue) and LA (red) evoked responses as percentage of longitudinal direction towards the MEC. The small dots represent the percentage for every experiment, the open circle represents the average ± standard error of the mean (SEM) over all tested slices. **(E)** Preference of the propagation in the perpendicular direction towards the white matter (WM) for AiP (blue) and LA (red) evoked responses as a percentage toward the WM. **(F,G)** Propagation velocity in the longitudinal **(F)** and perpendicular **(G)** direction for AiP (blue) and LA (red) evoked responses. For abbreviations see Figure 1.

Figure 4A shows a typical example of the propagation velocity field of AiP evoked activity. The next step in the spatial analysis was to decompose the velocity vector into components, with two main directions: towards MEC and AiP in the longitudinal direction (Figure 4C’, parallel to the pia) and towards the white matter (WM) and pia in the perpendicular direction (perpendicular to the pia—Figure 4C”). The percentage of the decomposed vectors pointing in the direction of the MEC or WM was plotted for PER, LEC and MEC (Figures 4D,E, respectively).

AiP stimulation evoked responses in the PER and LEC that propagated mainly in the rostral-to-caudal direction, preferentially towards the MEC (Figures 4B,D). In the perpendicular direction, AiP evoked responses preferentially propagated towards the WM in the PER/EC network (Figure 4E). LA evoked activity propagation in the longitudinal direction showed no clear preference in the PER and a preference towards the more caudal areas in the LEC (Figure 4D). Furthermore, there was a clear propagation preference in the perpendicular direction in the PER and LEC from the deep-to-superficial layers (Figure 4E). In the MEC, AiP and LA evoked responses showed a comparable propagation direction in the longitudinal direction towards the caudal MEC. The AiP evoked a response in the perpendicular direction towards the WM, while the LA there was no preference in the MEC (Figures 4D,E).

We calculated the main velocity for all the components in the longitudinal (Figure 4F) and perpendicular direction (Figure 4G) per subregion (averaging over all diodes within the subregion). The velocity in the longitudinal and perpendicular direction in response to AiP stimulation reduced from PER to MEC, while the velocity was comparable in all subregions in response to LA stimulation. The propagation velocities in the MEC were low, between 0.02 and 0.03 m/s, in both the longitudinal and perpendicular direction, for AiP as well as LA stimulation (Figures 4F,G).

The propagation pattern of AiP—evoked responses in the PER and the LEC was directed from rostral to caudal, whereas LA evoked responses propagated from the deep to superficial layers. In the MEC, AiP and LA stimulation evoked responses that propagated in the same direction.

### Primary Projection Site of AiP and LA Afferents in the PER/EC Network

The results described above showed that the PER/EC network is recruited after both AiP and LA stimulation in the presence of 1 μM bicuculline. However, the question remains how projections from the AiP and LA to the PER/EC network are organized. To examine the primary projection site for AiP and LA efferents to the PER/EC network, DiI neuronal tracer was injected in 400 μm slices (*n* = 4). AiP injection resulted in localized cortical staining of tissue in the superficial layers of the AiP (Supplementary Figure S2A). After DiI injection in LA, a projection to the deep layers of the PER was observed (Supplementary Figure S2B).

**Figure 5:**
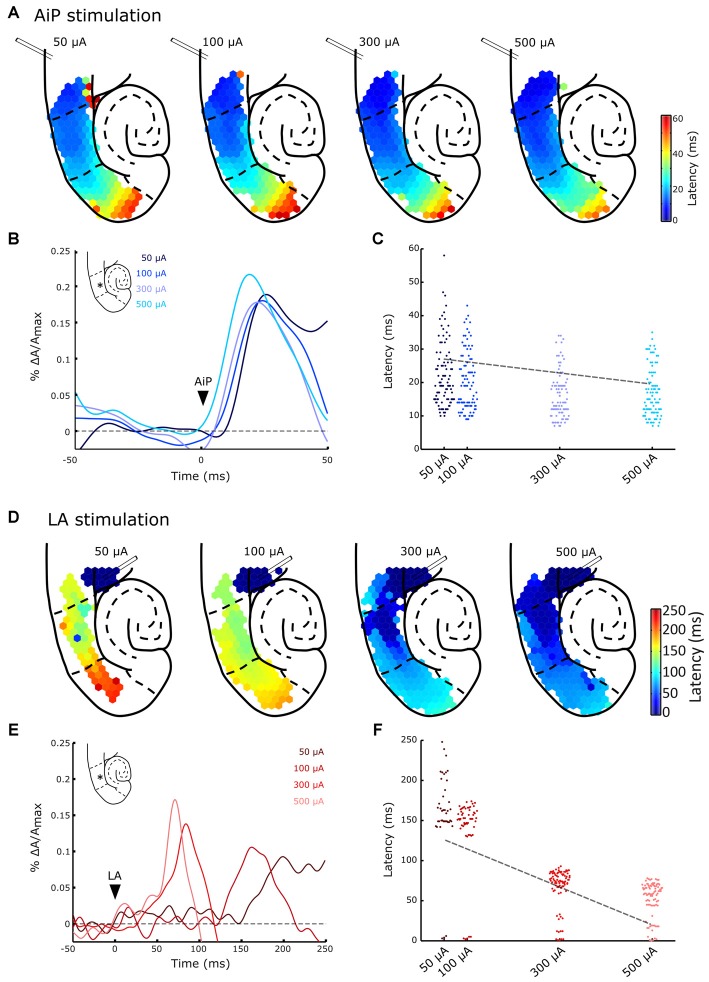
**Typical example of evoked responses after AiP (A,B) and LA (D,E) stimulation at four intensities (50, 100, 300 and 500 μA) in the presence of 1 μM bicuculline. (A)** Plots of the response latency distribution after AiP stimulation at the four increasing stimulus intensities (bins of 1 ms). **(B)** Temporal pattern of the AiP evoked response at four stimulus intensities recorded in the LEC at the channel indicated with an asterisk (*) in the inset. The arrowhead indicates the moment of stimulation. **(C)** Distribution plot of the latencies at all channels in the PER/entorhinal cortex (EC) network in the typical example represented in **(A)**, in response to AiP stimulation as a function of stimulus intensity. The data is fitted with a regression line (dotted line). **(D)** Plots of the response latency distribution after LA stimulation at four intensities (bins of 1 ms). Note the large color bar scale (0–250 ms) compared to the AiP evoked responses (**A**, 0–60 ms). **(E)** Temporal pattern of LA evoked response at four stimulus intensities recorded in the LEC at the channel indicated with an asterisk (*) in the inset. **(F)** Distribution plot of the latencies at all channels in the PER/EC network in response to LA stimulation as recorded in the typical example of **(D)** as a function of stimulus intensity. The data is fitted with a regression line (dotted line).

It was expected that regions primarily receiving the projection from the AiP or LA would respond to low stimulation intensities, whereas increasing the intensity would increase the recruited area. Therefore, the region showing responses after low intensity AiP and LA stimulation was examined in the control condition to determine which area was initially recruited in the PER/EC network by AiP or LA stimulation. Low intensity stimulation (50 μA) of the AiP in the control condition recruited the superficial and deep layers of the PER and LEC only in 3/9 slices (Supplementary Figures S1A,B). Once the network was recruited (3/9 slices), the shape of the response remained generally the same (Supplementary Figure S1B). MEC responses were never observed. When 1 μM bicuculline was applied, 50 μA AiP stimulation resulted in recruitment of the PER/EC network in 9/11 slices (Figure 5A).

Low intensity LA stimulation (50 μA) in the control situation (Supplementary Figures S1C,D) only recruited a small area of the PER/EC network in six out of nine slices: the activity was mainly observed in the PER deep layers (Supplementary Figure S1C), in line with the DiI staining observed in the PER deep layers (Supplementary Figure S2B). After application of 1 μM bicuculline, activity evoked by both low and high intensity stimulation spread towards superficial layers of the PER and MEC (Figure 5D).

### Increasing Stimulus Intensity in the LA Decreases the Response Latency in the PER and EC

To address the effect of increasing stimulus intensity on the latency and the spatial spread of evoked responses the AiP and LA were stimulated at four different intensities (i.e., 50, 100, 300 and 500 μA). Figure 5A shows a typical example of the spatiotemporal pattern of evoked responses at increasing stimulation intensities in the presence of bicuculline. Low intensity AiP stimulation (i.e., 50 and 100 μA, Figure 5A) already evoked responses above threshold in the PER/EC network in 9/11 slices. Stimulation at higher intensities evoked responses in all slices (11/11 slices, Figure 5A). AiP evoked activity propagated to the MEC at all tested stimulus intensities.

The typical responses to AiP stimulation at a specific channel in the LEC (Figure 5B, inset) are presented for the four tested intensities (Figure 5B). The shape of the response is comparable across intensities, so the latency response could be compared to examine the relation between latency and stimulation intensity. Figure 5C shows the latency of all channels in the PER/EC network at the four different stimulation intensities of the experiment shown in Figure 5A. Linear regression analysis was performed to reveal a possible relation between the stimulus intensity and the latency in the PER/EC network. If the slice responded to all four stimulus intensities (9/11 slices), increasing the AiP stimulation intensity was related to a decrease in the latency of responses in the PER/EC network (Figures 5B,C; Regression analysis: slope = −0.05 ± 0.05, *t*_(8)_ = −3.09, *p* = 0.01).

**Figure 6:**
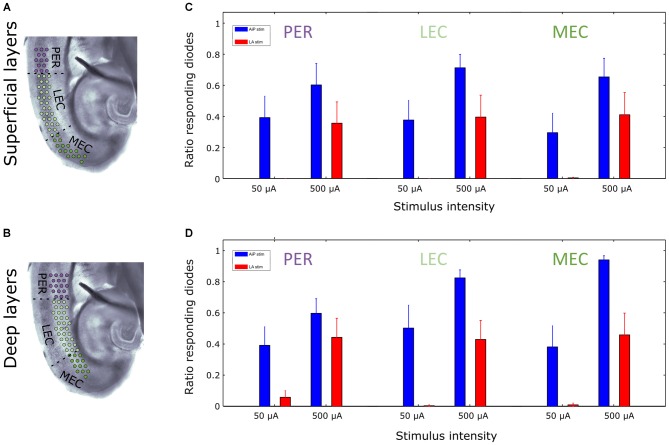
**Extent of activated region at two stimulation intensities in the superficial and deep layers of the PER/EC network in the presence of 1 μM bicuculline. (A,B)** Schematic overview of the analyzed subregions of the PER/EC network in the superficial **(A)** and deep **(B)** layers. **(C,D)** The fraction of diode channels detecting a response in a subregion after 50 μA and 500 μA AiP (blue) or LA (red) stimulation was calculated for the superficial **(C)** and deep **(D)** layers of the PER/EC network for all slices (*n* = 11).

Furthermore, the extent of the region showing responses was determined by calculating the number of channels showing evoked responses relative to the total number of channels covering the superficial or deep layers of the PER, LEC and MEC (Figures 6A,B). AiP stimulation (Figures 6C,D), either low (50 μA) or high (500 μA) intensities, always recruited a similar sized area of the PER/EC network in the superficial as well as in the deep layers (Figures 5A, 6C,D).

Figures 5D–F display an example of the same slice showing LA evoked responses to all four stimulation intensities. LA stimulation at 50 μA evoked a response in the PER/EC network in 5/11 slices. With increasing the stimulus intensity (100–500 μA) the PER/EC network responded in 9/11 slices to LA stimulation and the latency of LA evoked responses in the PER/EC network strongly decreased (Figures 5E,F). Regression analysis on the latency of all responses in the PER/EC network over all slices revealed a linear relationship between increasing stimulation intensities and a decrease in the latency of responses (Figure 5F; Regression analysis: slope = −0.211 ± 0.002, *t*_(4)_ = −6.59, *p* = 0.003).

The ratio of responding channels after LA stimulation was determined at low (50 μA) and high (500 μA) intensities. Stimulation of the LA at low stimulus intensities primarily activated the deep layers of the PER and LEC and subsequently the deep layers of the MEC. Higher stimulus intensities recruited the superficial layers of the PER, LEC, and MEC as well (Figures 5D, 6C,D). In the five slices that showed responses to all LA stimulation intensities, the size of the recruited area increased with increasing stimulus intensity in both deep and superficial layers (Figures 5D, 6C,D).

It took more time for LA stimulation to recruit the PER/EC network compared to AiP stimulation for all intensities and the network recruitment delay decreased with increasing LA stimulation intensity. The threshold for PER/EC network recruitment was lower for the AiP than the LA afferents, suggesting more efficient recruitment of the PER/EC network by its AiP afferents.

## Discussion

AiP and LA projections differently target the PER/EC network. The AiP has direct axonal projections primarily to the PER superficial layers (Mathiasen et al., 2015). In contrast, the LA projects to both deep and superficial layers of the PER and LEC (Pitkänen et al., 2000; Sparta et al., 2014). These distinct projections are hypothesized to recruit the PER/EC network in a different fashion. Using VSD imaging, we recorded stimulus evoked activity in the PER/EC network and this technique provided the opportunity to analyze subregion, and cortical layer specific recruitment of the PER/EC network. A different spatiotemporal activation pattern of the mouse PER/EC network was revealed in response to AiP and LA stimulation. Furthermore, the results that the inhibitory circuitry can regulate the functional recruitment of the PER/EC network by its insular and amygdala afferents. We used the competitive GABA_A_ antagonist bicuculline to reduce the inhibition in this study. The concentration used (1 μM) reduces the GABAergic inhibition, by blocking approximately 20% of the GABA_A_ dependent chloride conductance (Alger and Nicoll, 1982). This concentration has never shown to induce epileptiform activity (Iijima et al., 1996; Menendez de la Prida and Pozo, 2002).

### Sequential PER and EC Network Recruitment in Response to AiP Stimulation

First, we determined which subarea of the PER/EC network was primarily recruited after stimulation. In the control condition, AiP stimulation at low intensities recruited first the PER and subsequently the LEC in 3/9 slices. This was corroborated by the recruitment pattern after high intensity stimulation, in which the PER was the first area to show evoked activity in all slices. Likewise, Pelletier et al. (2004) showed that neocortical stimulation was more effective in activating PER neurons compared to EC neurons in the *in vivo* cat brain. Based on anatomical evidence, these PER responses may result from both mono- and polysynaptic connections (Mathiasen et al., 2015). DiI injections in the AiP primarily labeled axons close to the injections site, indicating that the evoked activity in the PER/EC network in this study is mainly of a polysynaptic origin.

Reducing GABAergic inhibition changed both the spatial and temporal aspect of the propagation pattern evoked by AiP stimulation. Detailed evaluation of the propagation pattern in the presence of bicuculline showed that, following the initial PER activity, the LEC showed responses. This is most likely accomplished by polysynaptic projections from the AiP via the PER superficial layers to the LEC (Burwell and Amaral, 1998; Biella et al., 2001; Martina et al., 2001b; de Villers-Sidani et al., 2004; van Strien et al., 2009). The MEC was eventually only recruited during the partial block of GABA_A_ receptors. The latency of this MEC activation was longer than that for the PER and LEC, in line with the polysynaptic intrinsic EC projections (Lavenex and Amaral, 2000; Canto et al., 2008) and possible a strong influence of inhibitory neurons on excitation in the MEC network (Jones and Bühl, 1993).

Besides the longitudinal propagation from the PER to the MEC in response to AiP stimulation, there was a perpendicular recruitment from the superficial toward the deep layers in the PER/EC network, which is hypothesized to reflect cortical columnar processing (Hirata and Sawaguchi, 2008; Wester and Contreras, 2012). Corresponding to this hypothesis, this study showed that both superficial and deep layers of the PER/EC network were both recruited after AiP stimulation. The superficial-to-deep spread in PER could be explained by the projection from the layers II/III to layer V pyramidal cells (Markram et al., 1997). The layer V activity could create a horizontal spread of excitation over the cortical network (Tanifuji et al., 1994; Wester and Contreras, 2012).

The propagation velocities determined in PER and LEC are comparable with those reported in neocortical slices, where they are suggested to reflect sequential synaptic activation of interconnected adjacent neurons (Wadman and Gutnick, 1993; Wu et al., 2008; Sato et al., 2012). In light of these findings, the results presented here suggest a columnar recruitment of the PER/EC network in response to AiP stimulation. The velocity decreased in the MEC, probably indicating the involvement of polysynaptic network recruitment involving a balanced interplay between excitation and inhibition in the MEC (Biella et al., 2002). This polysynaptic propagation of activity in the MEC could result in a less synchronized and therefore more time consuming recruitment of the network.

### Delayed Recruitment of the PER/EC Network After LA Stimulation

LA stimulation evoked a response restricted to the LA and a small portion of the PER deep layers in the control condition. This observation was confirmed by DiI labeling located in the PER deep layers. When inhibition was reduced, the deep layers of the PER and subsequently the deep layers of the LEC were recruited after LA stimulation. Anatomical tracer studies *in vivo* described that fibers from the amygdala terminate in all layers of the PER and EC. While the PER afferents are mainly glutamatergic, the EC receives both glutamatergic and GABAergic input (Krettek and Price, 1977; Smith and Paré, 1994; Petrovich et al., 1996; Pitkänen et al., 2000; McDonald and Zaric, 2015; McDonald and Mott, 2016), suggesting that relieve of inhibition results in recruitment of the EC network.

Increasing the LA stimulus intensity during reduced GABAergic inhibition, increased the efficiency of the stimulus: after activation of the deep layers of the PER/EC, their corresponding superficial layers were recruited as well. Projections from the LA to the PER can uniformly activate the PER along the rostrocaudal axis (Pelletier and Paré, 2002), with a large variation in latency (ranging between 2–27 ms) but unrelated to the distance between the PER and the LA. This uniformity could be achieved by adjustment of the conduction velocity (i.e., by differences in myelination and/or axonal thickness) depending on the axon length as seen for instance in the olivocerebellar pathway (Lang and Rosenbluth, 2003). Uniform recruitment can be advantageous for creating synchrony between brain areas.

LA evoked response latencies were larger than those of AiP evoked responses, although comparable to the delays of 30–60 ms found in VSD imaging studies in acute rodent brain slices (Kajiwara et al., 2003; Koganezawa et al., 2008; Biella et al., 2010). The strikingly long delays for PER/EC network recruitment can be explained by various processes. First, LA projections to the PER are known to be less dense than neocortical PER afferents. Stimulation of the neocortex results in firing of 42% of PER cells *in vivo* (Pelletier et al., 2004, 2005), while LA stimulation orthodromically only activates 15% of the PER neurons (Pelletier and Paré, 2002; Pelletier et al., 2005). The weak connectivity from the LA towards the PER/EC network could result in slowly developing activity in the PER/EC local deep layer network. The weak input may recruit a small subset of deep layer neurons, which polysynaptically recruit the local PER and EC network. Only once a significant population of the PER/EC network is recruited, VSD responses will be detected by the photodiode array. This hypothesis is supported by the decreased ratio of channels showing responses in the PER/EC network after low intensity LA stimulation. The chance of successful synaptic transmission decreases with decreased stimulus intensity and therefore can therefore be a predictor for polysynaptic network recruitment (Bailey et al., 2006). Second, the long delays of LA evoked responses may be explained by the occurrence of late-spiking (LS) neurons in the LA and in the PER, which can delay their firing upon stimulation (Faulkner and Brown, 1999; Beggs et al., 2000; McGann et al., 2001; Moyer et al., 2002) and therefore the local recruitment of the PER/EC network is possibly postponed.

### Comparison of the AiP and LA Stimulus Induced PER/EC Recruitment Patterns

In conclusion, this study presented the spatiotemporal pattern of PER/EC network recruitment after stimulation of its AiP or LA afferents. We observed that the AiP initially recruits the PER superficial layers, whereas the LA first recruits the PER deep layers. The EC network is recruited with a delay. However, both inputs eventually recruit the PER/EC network.

We thus propose that both inputs at least partly share the same polysynaptic network. This hypothesis is supported by work of Pelletier et al. (2005). They showed that stimulation of the temporal neocortex or the LA both evoke synaptic responses in PER and EC neurons, but that the excitatory and inhibitory components depend more on the properties of the targeted neuron, rather than on which region is stimulated. However, it is not yet clear whether the neocortex and LA directly project to the same cells or whether these inputs project both to their own subset of neurons. Furthermore, Koganezawa et al. (2008) showed that LA stimulation could potentiate activity in the EC deep layers evoked by PER stimulation. The findings that the AiP and LA share a polysynaptic network, indicates a possible modulatory role for the LA in AiP evoked activity in the PER/EC. However, the delay after LA stimulation indicated that the LA activation has to precede incoming neocortical input in the PER/EC network for emotional loading of neocortical information before it enters the memory consolidation circuitry.

### Proposed Mechanism for PER/EC Recruitment by the Lateral Amygdala and Insular Activity

The role of the PER/EC network in the regulation of impulse traffic from neocortical areas towards the hippocampus for memory storage has been widely discussed (for review, see de Curtis and Paré, 2004). There are several reports of low probability transmission of cortical or PER evoked activity to the EC (Biella et al., 2003; Kajiwara et al., 2003; Pelletier et al., 2004; Koganezawa et al., 2008) corroborating the absence of activity propagation towards the MEC in the control condition in the present study. Furthermore, we showed that AiP evoked activity in the PER can propagate towards the LEC and MEC in acute mouse brain slices in the presence of a low concentrations of a competitive GABA_A_ antagonist. We propose that short- and long-range GABAergic interneurons can regulate transfer of evoked response through the PER/EC network, even entirely into the MEC (Pinto et al., 2006). Martina et al. (2001b) observed that electrical stimulation in the neocortex, adjacent to the PER recording site, generated both inhibitory and excitatory synaptic potentials in PER neurons, while electrical stimulation at more distant sites induced pure excitatory responses. Therefore, the reduction in inhibition in our experiment may have several consequences. First, a system of long-range GABAergic feed-forward projections is reported in the PER/EC network, consisting of long-range GABAergic projections from the neocortex to the PER and EC and from the PER projection to the EC (Pinto et al., 2006; Apergis-Schoute et al., 2007). Second, recruitment of PER neurons may recruit local inhibition, which is capable of impeding the propagation of excitation to subsequent areas (Samarth et al., 2016). Slightly diminishing the strength of the short-and long-range inhibition—as the concentration of bicuculline used in this study, only blocks 20% of the inhibitory GABA_A_ conductance (Alger and Nicoll, 1982)—may facilitate the information transfer through the PER/EC, so excitation can travel over the PER/EC network without being hindered.

Our results suggest that the information propagation through the PER/EC network is promoted when inhibition is reduced. In behavioral states, this inhibitory control of excitation could regulate information transfer. Once input from various brain areas can decrease this inhibitory block or enhance excitation, for example via neuromodulatory activity (Apergis-Schoute et al., 2007), the probability of information transfer to the upstream areas, for instance the hippocampus, could be increased.

## Author Contributions

JGPW and NLMC designed the experiment, JGPW and NLMC performed the experiment and analysis, JGPW, WJW and NLMC interpreted data, JGPW and NLMC wrote the manuscript, JGPW, WJW and NLMC discussed and reviewed the manuscript.

## Conflict of Interest Statement

The authors declare that the research was conducted in the absence of any commercial or financial relationships that could be construed as a potential conflict of interest.

## Supplementary Material

Figure S1 **Example of evoked activity in the PER/EC network in response to 50 μA stimulation in AiP (A,B) and LA (C,D) in the control condition.** Plots of the latency distribution after 50 μA stimulation of the AiP (**A**—bins of 1 ms) and LA (**C**—bins of 1 ms). **(B)** Temporal pattern of AiP evoked response at a channel in the superficial layers of the PER indicated with an asterisk (*) in the inset. **(D)** Distribution of LA evoked response latencies at four stimulus intensities recorded at a channel in the deep layers of the PER indicated with an asterisk (*) in the inset. The arrowhead indicates the moment of stimulation.

Figure S2 **Example of DiI (red) injected slices. (A)** DiI injection in the AiP in a 400 μm slice resulted in localized staining around the injection side **(A’)**. The inset shows the schematic overview of the injection side. **(B)** DiI injection in the LA resulted in complete staining of the LA. Furthermore, DiI staining was found in the deep layers of the PER **(B’)**. The inset shows the schematic overview of the injection side. Cell nuclei were counterstained with DAPI (blue).
