# Supplementary figures and images for: Distinct Spatiotemporal Activation Patterns of the Perirhinal-Entorhinal Network in Response to Cortical and Amygdala Input
Source: Front Neural Circuits. 2016 Jun 14;10:44. doi: 10.3389/fncir.2016.00044 (PMC4906015; doi:10.3389/fncir.2016.00044)

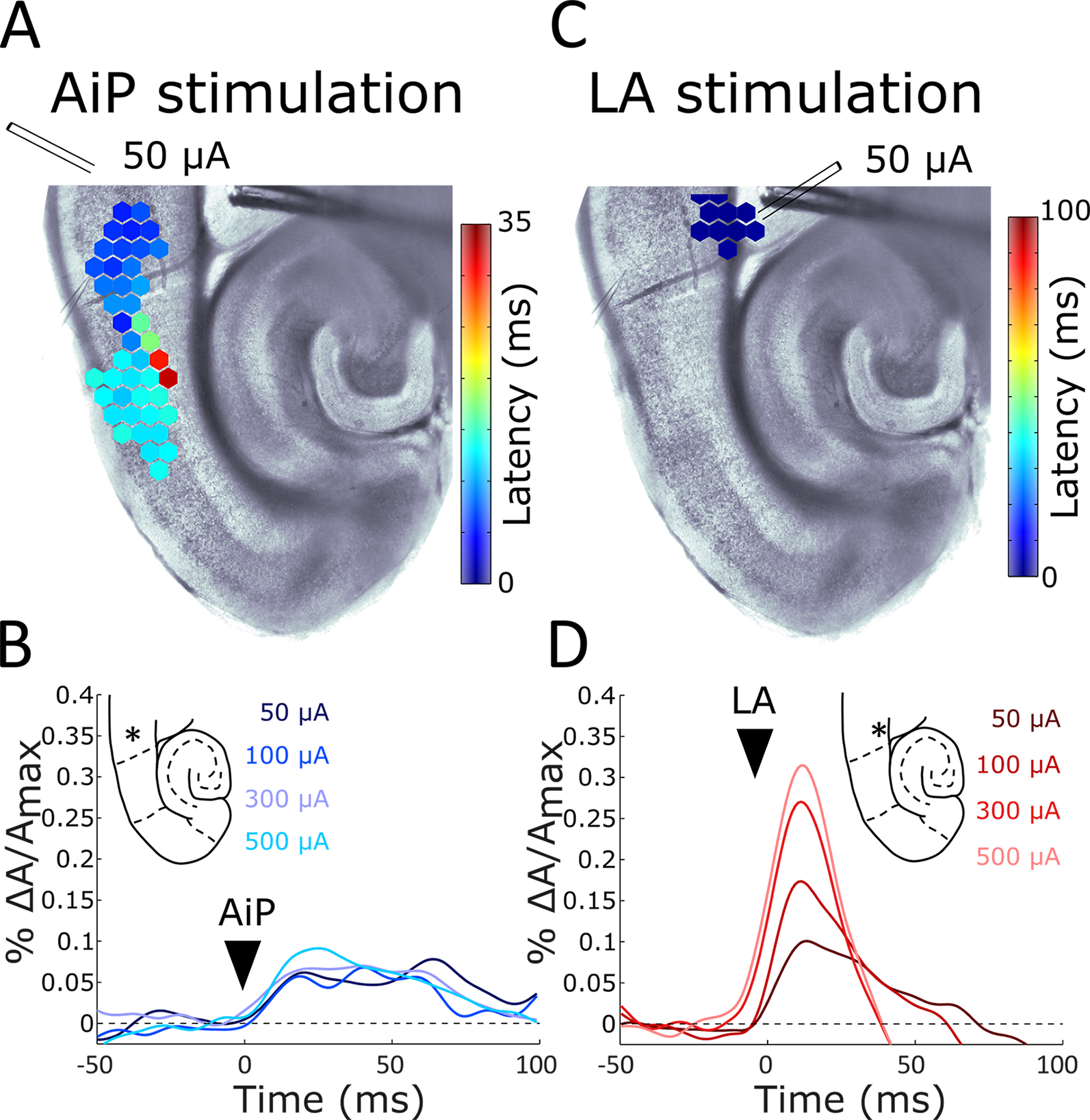

Supplement: Figure S1 — Example of evoked activity in the PER/EC network in response to 50 μA stimulation in AiP (A,B) and LA (C,D) in the control condition. Plots of the latency distribution after 50 μA stimulation of the AiP (A—bins of 1 ms) and LA (C—bins of 1 ms). (B) Temporal pattern of AiP evoked response at a channel in the superficial layers of the PER indicated with an asterisk (*) in the inset. (D) Distribution of LA evoked response latencies at four stimulus intensities recorded at a channel in the deep layers of the PER indicated with an asterisk (*) in the inset. The arrowhead indicates the moment of stimulation. [file Image_1.tif]

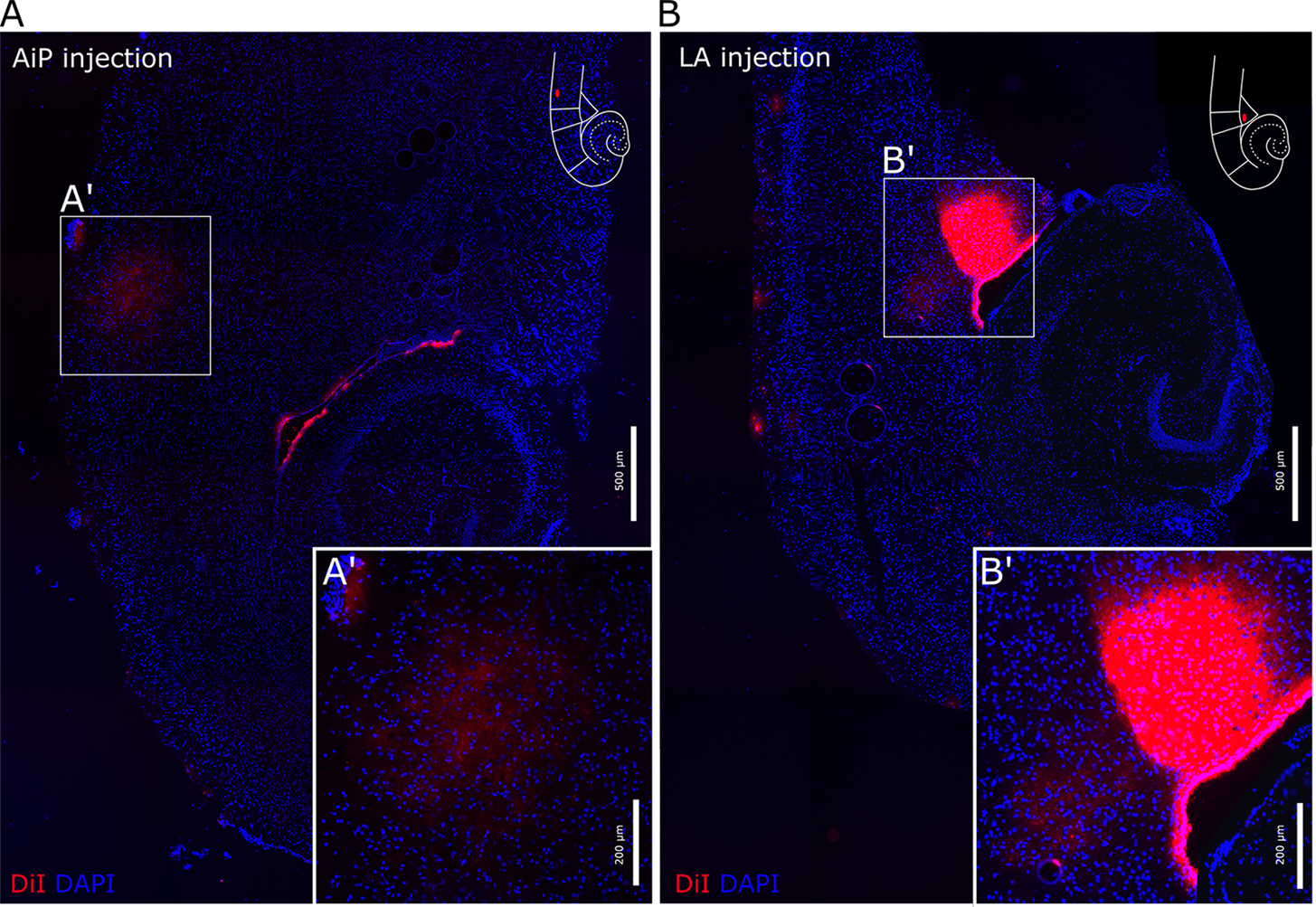

Supplement: Figure S2 — Example of DiI (red) injected slices. (A) DiI injection in the AiP in a 400 μm slice resulted in localized staining around the injection side (A’). The inset shows the schematic overview of the injection side. (B) DiI injection in the LA resulted in complete staining of the LA. Furthermore, DiI staining was found in the deep layers of the PER (B’). The inset shows the schematic overview of the injection side. Cell nuclei were counterstained with DAPI (blue). [file Image_2.tif]
